# Anti-Sway and Positioning Adaptive Control of a Double-Pendulum Effect Crane System With Neural Network Compensation

**DOI:** 10.3389/frobt.2021.639734

**Published:** 2021-04-19

**Authors:** Hai-yan Qiang, You-gang Sun, Jin-chao Lyu, Da-shan Dong

**Affiliations:** ^1^ College of Logistics Engineering, Shanghai Maritime University, Shanghai, China; ^2^ Institute of Rail Transit, Tongji University, Shanghai, China

**Keywords:** double-pendulum, neural network, adaptive control, anti-sway and positioning, hardware in the loop

## Abstract

Cranes are widely used in the field of construction, logistics, and the manufacturing industry. Cranes that use wire ropes as the main lifting mechanism are deeply troubled by the swaying of heavy objects, which seriously restricts the working efficiency of the crane and even cause accidents. Compared with the single-pendulum crane, the double-pendulum effect crane model has stronger nonlinearity, and its controller design is challenging. In this paper, cranes with a double-pendulum effect are considered, and their nonlinear dynamical models are established. Then, a controller based on the radial basis function (RBF) neural network compensation adaptive method is designed, and a stability analysis is also presented. Finally, the hardware-in-the-loop experimental results show that the neural network compensation control can effectively improve the control performance of the controller in practice.

## Introduction

Cranes are normally utilized to lift or move cargo in the field of construction, logistics, the manufacturing industry, etc. Cranes come in a variety of forms, and most cranes work by utilizing wire ropes to suspend cargo. With crane motion or external disturbances, cargo will sway owing to the flexibility of the wire ropes, and active and passive methods are taken to carry the cargo to the desired position. The swaying of the cargo is a kind of simple harmonic vibration. When the cargo carried by the cranes begins to sway, it needs several periods to be suppressed, which is time-consuming and even lead to working efficiency problems. Therefore, the swaying problem has become an urgent subject, especially for the port industry. More than that, the swaying problem will cause safety problems when carrying cargo of great mass, and it may even influence the design of the crane structure greatly.

Anti-swaying technology for cranes has been studied in the last 20 years. Attention was paid to both single-pendulum and double-pendulum effect cranes by using mechanical and automatic anti-swaying technology. When the mass of the spreader for the crane is big enough, the double-pendulum effect of the cranes cannot be neglected. In order to eliminate this double-pendulum effect, which brings stronger nonlinearity caused by interactions between the spreader and the payload into the system, controllers are designed to handle the swaying. A lot of research has been done to design such controllers for solving the double-pendulum effect. Kamal (Moustafa and Ebeid, 1988) et al. derived the nonlinear dynamical model of cranes, and, in 1988, a feedback control method was also presented to eliminate the swaying of the cargo. M. Guitierrez et al. (Gutierrez and Solo, 1998) proposed a fuzzy logic controller for an overhead crane prototype model that had obtained excellent control performances in simulations and experiments. Z Nowacki et al. (Nowacki et al., 1996) presented a PD controller with two feedback loops for an overhead crane and robustness analysis of the system was also proposed. Chwa et al. (Dongkyoung, 2017) presented a tracking control method based on a sliding mode control method for a three-dimensional overhead crane system to suppress the sway angels of the trolley, and the control performance was proved to be excellent. H.H Lee et al. (Cho and Lee, 2002) presented a novel fuzzy anti-swing control scheme combined with a position servo control and fuzzy logic control for an overhead crane prototype and the effectiveness of the proposed control strategy is proved by experiments.

Owing to the excellent approximation capability of the neural network method, researchers have presented several studies to solve the swaying problem of the cranes. Chunshien Li and Chun-yi Lee et al. (Li and Lee, 2001a; Li and Lee, 2001b) proposed a composite method combined with a neural network adaptive control method with fuzzy control for an overhead crane with high capacity to resist disturbance. Park et al. (Park and Le, 2012) had proposed a virtual prototype co-simulation by MATLAB and ADAMS to study the floating container crane. Zhang et al. (Zhang et al., 2016) proposed an adaptive tracking controller based on double-pendulum overhead cranes with uncertainties and disturbances by building a new sliding function as the desired trajectory. Ouyang et al. (Ouyang et al., 2019) presented a novel adaptive hierarchical sliding mode controller for overhead cranes with a double-pendulum effect, which can make the states of the system enter the desired sliding surface faster and even improve the precision of cart tracking. Ning Sun et al. (Sun et al., 2020a) established a dynamic model of a cooperative dual rotary crane system based on Lagrange’s method. Considering the actuator constraints, an output feedback control method that helps to increase the dual-boom positioning accuracy was also presented. Also, the control method mentioned in (Sun et al., 2020a) was extended to the positioning and tracking control for a pneumatic artificial muscle system (Sun et al., 2020b). Chen H et al. presented a new control strategy for a kind of under-actuated system by treating different constraints, and the proposed method was applied to a double-pendulum crane system with superior performances (Chen et al., 2019; Chen and Sun, 2020). Yang T et al. (Yang et al., 2020) designed an adaptive control method based on the neural network to solve the positioning problems for a ship-mounted crane system.

Based on the previous analysis, the elimination of the double-pendulum effect of cranes plays an important role to ensure the safety and effectiveness of cranes during operation. The main contribution of this work is as follows:

1. The proposed controller can realize fast positioning and anti-swing function for the crane system with a double swing effect.
2. The adaptive controller based on RBF neural network compensation has an online learning function and is not sensitive to the change of parameters. Even if there are some missing items in the controller, it can still get a better control effect through online learning compensation.
3. Experiment results show that the positioning accuracy and response speed are better than the traditional sliding mode controller.

In this paper, an overhead crane with a double-pendulum effect is chosen as the research object. Firstly, the mathematical model of the double-pendulum effect crane is established. Base on the mathematical model, an adaptive controller based on a radial basis function neural network compensation method is designed where the core of the controller is the robust controller, and the neural network is worked as an additional system to output compensation force. Then, the Lyapunov stability analysis is performed to testify the stability of the neural network controller crane system. Finally, hardware in a loop experimental platform is utilized to verify the proposed controller. A comparison between the sliding mode controller and the neural network controller is also presented to verify the effectiveness and feasibility of the control strategy.

## Modeling of Double-Pendulum Effect Cranes

**FIGURE 1:**
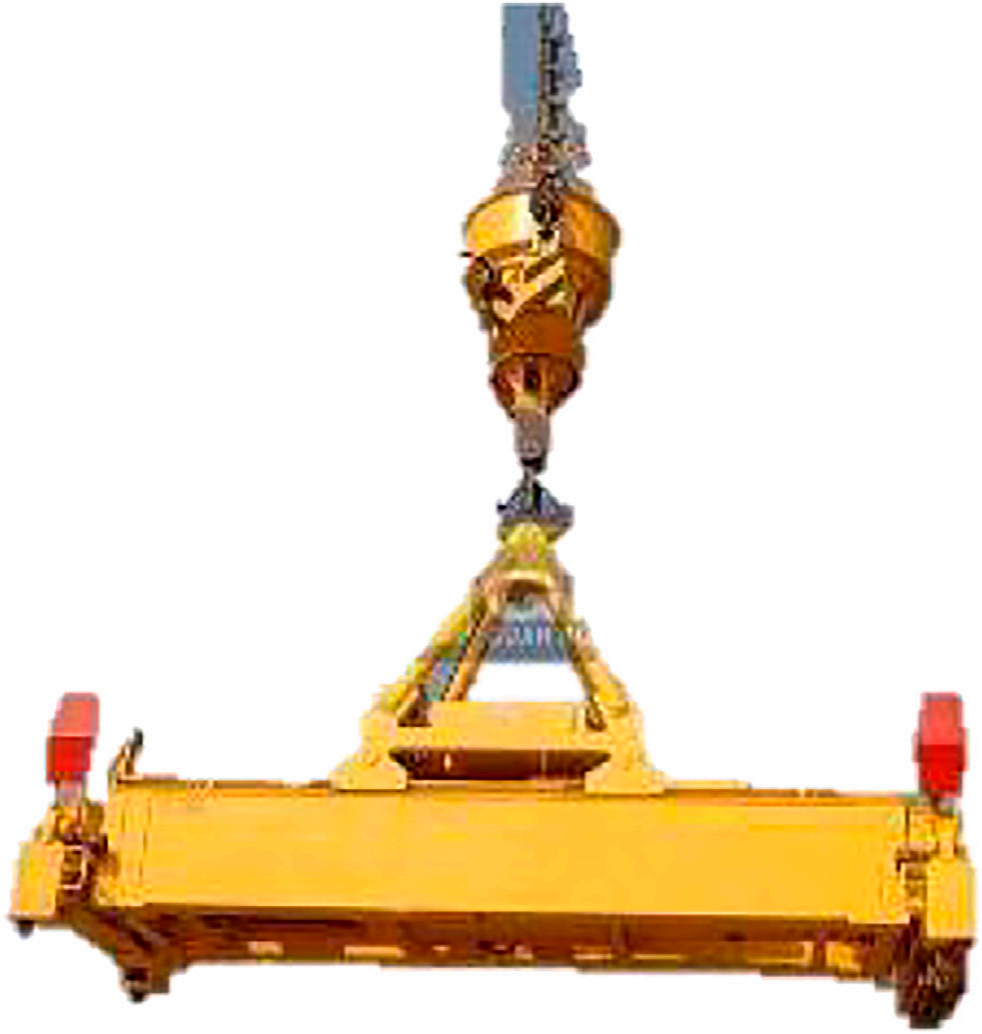
Double-pendulum crane.

The structure of the overhead crane with a double-pendulum effect is composed of a trolley, a spreader with a large mass (first-order payload), and a payload (second-order payload). Connections between the spreader, trolley, and payload are made of wire ropes, which are shown in Figure 1.

**FIGURE 2:**
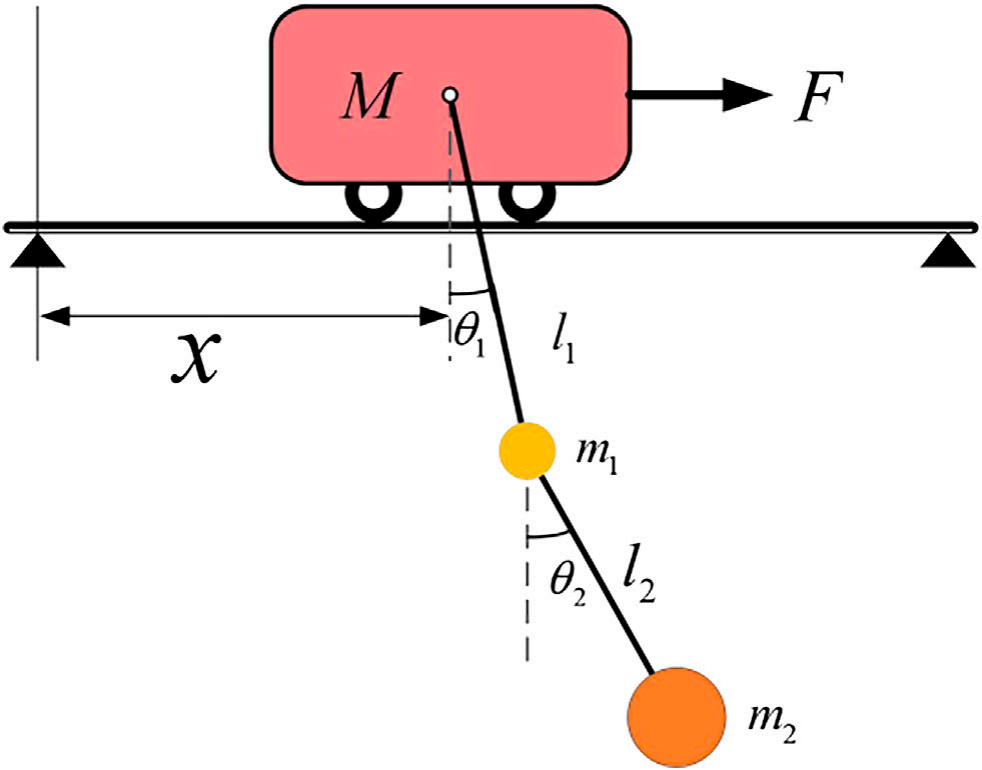
Simplified model of the crane with double-pendulum.

This assumes that the trolley, spreader, and payload of the crane are working on a two-dimensional plane, and the track of the trolley is horizontal. It considers the spreader and payload as particles, and the connection point of the wire rope is on the centroid of the spreader and payload. The transient wind disturbances and friction among the trolley, spreader, and payload connection point are ignored. The simplified model is shown in Figure 2.

In Figure 2, the positive direction for the *x* axis is pointed to the right; *F* denotes the driving force of the trolley; *f* represents the friction force between the trolley and the track; *M* denotes the mass of the trolley; *m*_1_ and *m*_2_ represent the mass of the spreader and the payload, respectively; *l*_1_ and *l*_2_ denote the wire rope length among the centroid of the spreader, trolley, and the payload, respectively; *x* represents the displacement of the trolley; *θ*_1_ and *θ*_2_ denote the swing angle of the spreader and payload, respectively.

According to Lagrange equation of the second kind, the dynamic equation set of the crane with double-pendulum can be obtained:

$$\{\begin{array}{l} \begin{array}{l} \left(M+m_{1}+m_{2}\right)\ddot{x}+\left(m_{1}+m_{2}\right)l_{1}\left({\ddot{\theta }}_{1}\cos\theta_{1}-{\dot{\theta }}_{1}^{2}\sin\theta_{1}\right)\\ +m_{2}l_{2}\left({\ddot{\theta }}_{2}\cos\theta_{2}-{\dot{\theta }}_{2}^{2}\sin\theta_{2}\right)+b\dot{x}=F \end{array}\\ \begin{array}{l} m_{1}l_{1}\left(\ddot{x}\cos\theta_{1}+g\sin\theta_{1}+l_{1}{\ddot{\theta }}_{1}\right)+m_{2}l_{1}[l_{1}{\ddot{\theta }}_{1}+l_{2}{\ddot{\theta }}_{2}\cos\left(\theta_{1}-\theta_{2}\right)\\ +l_{2}{\dot{\theta }}_{2}^{2}\sin\left(\theta_{1}-\theta_{2}\right)+\ddot{x}\cos\theta_{1}+g\sin\theta_{1}]=0 \end{array}\\ \begin{array}{l} m_{2}l_{2}^{2}{\ddot{\theta }}_{2}+m_{2}l_{1}l_{2}{\ddot{\theta }}_{1}\cos\left(\theta_{1}-\theta_{2}\right)-m_{2}l_{1}l_{2}{\dot{\theta }}_{1}^{2}\sin\left(\theta_{1}-\theta_{2}\right)\\ +m_{2}l_{2}\ddot{x}\cos\theta_{2}+m_{2}l_{2}g\sin\theta_{2}=0 \end{array} \end{array}$$ (1)

where *b* denotes the damping coefficient; $\dot{x}$ represents the velocity of the trolley; $\ddot{x}$ denotes the acceleration of the trolley; ${\dot{\theta }}_{1}$ and ${\ddot{\theta }}_{1}$ represent the angular velocity and acceleration of the swing angle 1 respectively; ${\dot{\theta }}_{2}$ and ${\ddot{\theta }}_{2}$ denote the angular velocity and acceleration of the swing angle 2, respectively.

## Adaptive Controller Based on RBF Neural Network Compensation Method

Owing to the development of neural network technology (Hunt et al., 1992; Feng, 1995), a great process had been made in the field of automatic control, signal processing, and pattern recognition. In this case, the neural network method had been introduced into the crane system, and the model of the double-pendulum crane follows Eq. 2:

$$M\left(q\right)\ddot{q}+C\left(q,\dot{q}\right)\dot{q}+G\left(q\right)=\tau +d$$ (2)

where $q={\left[\begin{array}{ccc} x & \theta_{1} & \theta_{2} \end{array}\right]}^{T}$; $\dot{q}={\left[\begin{array}{ccc} \dot{x} & {\dot{\theta }}_{1} & {\dot{\theta }}_{2} \end{array}\right]}^{T}$; $\ddot{q}={\left[\begin{array}{ccc} \ddot{x} & {\ddot{\theta }}_{1} & {\ddot{\theta }}_{2} \end{array}\right]}^{T}$; $\tau ={\left[\begin{array}{ccc} F & 0 & 0 \end{array}\right]}^{T}$;

The control purpose of the double-pendulum crane is to help the trolley stop at the desired position while the first and second payloads which is set below the trolley keeps still. The tracking signal is defined as $q_{d}=\left[\begin{array}{c} x_{d}\\ \theta_{1d}\\ \theta_{2d} \end{array}\right]$; ${\dot{q}}_{d}=\left[\begin{array}{c} {\dot{x}}_{d}\\ {\dot{\theta }}_{1d}\\ {\dot{\theta }}_{2d} \end{array}\right]$; ${\ddot{q}}_{d}=\left[\begin{array}{c} {\ddot{x}}_{d}\\ {\ddot{\theta }}_{1d}\\ {\ddot{\theta }}_{2d} \end{array}\right]$.

**FIGURE 3:**
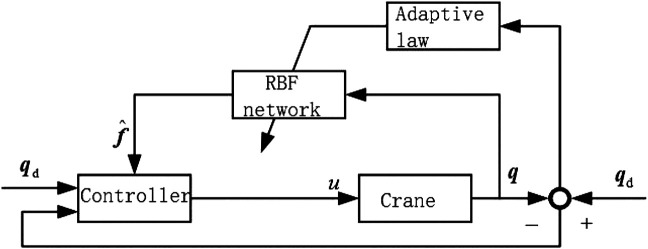
Adaptive control based on neural network compensation.

The calculated traction is utilized to control the double-pendulum crane system. The controller is composed of a traction calculation method and a neural network compensation method. An RBF neural network correction controller is defined as an additional to recognize the online model error of the crane. As shown in Figure 3.

### Calculated Traction Controller

The calculated traction control method is defined as a kind of robust controller (Weiping et al., 2004). According to the dynamic Eq. 2 of the crane, the control law can be designed as follows:

$$\tau =M_{0}\left(q\right)\left({\ddot{q}}_{d}-k_{v}\dot{e}-k_{p}e\right)+C_{0}\left(q,\dot{q}\right)\dot{q}+G_{0}\left(q\right)$$ (3)

where $k_{p}=\left[\begin{array}{ccc} \alpha_{1} & 0 & 0\\ 0 & \alpha_{2} & 0\\ 0 & 0 & \alpha_{3} \end{array}\right]$; $k_{v}=\left[\begin{array}{ccc} \beta_{1} & 0 & 0\\ 0 & \beta_{2} & 0\\ 0 & 0 & \beta_{3} \end{array}\right]$, α > 0, β > 0.

The systematic error is defined as follows:

$$\ddot{e}+k_{v}\dot{e}+k_{p}e=0$$ (4)

where $e=q-q_{d}$; $\dot{e}=\dot{q}-{\dot{q}}_{d}$; *q*_d_ represents the ideal tracking signal.

### RBF Neural Network Controller and Stability Analysis

RBF neural network method is adopted to approximate f(·) and the RBF network algorithm can be defined as follow:

$$h_{i}=\exp\left(\frac{{\left\|x-c_{i}\right\|}^{2}}{2b_{i}^{2}}\right),i=1,2,\ldots ,n$$ (5)

$$f=w^{T}h\left(x\right)$$ (6)

where the input of the network is defined as $x={\left[e,\dot{e}\right]}^{T}$; ***c***_*i*_ denotes the coordinate vector of the center point of the Gaussian basis function of the *i*th neuron in the hidden layer of the network; ***b***_*i*_ represents the width for Gaussian function of the *i*th neuron in the hidden layer; $h={\left[h_{1},h_{2},\ldots ,h_{n}\right]}^{T}$ denotes the calculated output of Gaussian function; w represents the weight of the network; ***f*** denotes the compensation force output from the network.

The adaptive control law can be designed as follows:

$$\dot{\hat{w}}=\gamma hx^{T}PB$$ (7)

where γ > 0; $A=\left(\begin{array}{cc} 0 & I\\ -k_{p} & -k_{v} \end{array}\right)$; $B=\left[\begin{array}{c} 0\\ M_{0}^{-1}\left(q\right) \end{array}\right]$.

The Lyapunov function is designed as follows:

$$V=\frac{1}{2}x^{T}Px+\frac{1}{2\gamma }{\left\|\tilde{w}\right\|}^{2}$$ (8)

where γ > 0.

The matrix ***P*** is Symmetric and positive definite. Moreover, ***P*** should satisfy the Lyapunov equation as follows:

$$PA+A^{T}P=-Q$$ (9)

where ***Q*** ≥ 0.

$${\left\|R\right\|}^{2}=\sum_{i,j}{\left|r_{ij}\right|}^{2}=tr\left(RR^{T}\right)=tr\left(R^{T}R\right)$$ (10)

where tr(·) denotes the trace of the matrix. We can obtain the following:

$${\left\|\tilde{w}\right\|}^{2}=tr\left({\tilde{w}}^{T}\tilde{w}\right)$$ (11)

***V*** is derived with respect to time, and we can get the following:

$$\begin{array}{l} \dot{V}=\frac{1}{2}\left[x^{T}P\dot{x}+{\dot{x}}^{T}Px\right]+\frac{1}{\gamma }tr\left({\dot{\tilde{w}}}^{T}\tilde{w}\right)\\ =\frac{1}{2}\left\{x^{T}P\left[Ax+B\left(\eta -{\tilde{w}}^{T}h\right)\right]+\left[x^{T}A^{T}+{\left(\eta -{\tilde{w}}^{T}h\right)}^{T}B^{T}\right]Px\right\}+\frac{1}{\gamma }tr\left({\dot{\tilde{w}}}^{T}\tilde{w}\right)\\ =\frac{1}{2}\left[x^{T}\left(PA+A^{T}P\right)x+\left(x^{T}PB\eta -x^{T}PB{\tilde{w}}^{T}h+\eta^{T}B^{T}Px-h^{T}\tilde{w}B^{T}Px\right)\right]+\frac{1}{\gamma }tr\left({\dot{\tilde{w}}}^{T}\tilde{w}\right)\\ =\frac{1}{2}x^{T}Qx+\eta^{T}B^{T}Px-h^{T}\tilde{w}B^{T}Px+\frac{1}{\gamma }tr\left({\dot{\tilde{w}}}^{T}\tilde{w}\right) \end{array}$$

where $x^{T}PB{\tilde{w}}^{T}h=h^{T}\tilde{w}B^{T}Px$; $x^{T}PB\eta =\eta^{T}B^{T}Px$.

Due to

$$h^{T}\tilde{w}B^{T}Px=tr\left[B^{T}Pxh^{T}\tilde{w}\right]$$ (12)

$\dot{V}$ can be rewritten as follows:

$$\begin{array}{l} \dot{V}=-\frac{1}{2}x^{T}Qx+\frac{1}{\gamma }tr\left(-\gamma B^{T}Pxh^{T}\tilde{w}+{\dot{\tilde{w}}}^{T}\tilde{w}\right)+\eta^{T}B^{T}Px\\ =-\frac{1}{2}x^{T}Qx+\eta^{T}B^{T}Px \end{array}$$ (13)

According to the known conditions, it can be concluded that $\left\|\eta^{T}\right\|\leq \left\|\eta_{0}\right\|$, $\left\|B\right\|=\left\|M_{0}^{-1}\left(q\right)\right\|$

$$\begin{array}{l} \dot{V}\leq -\frac{1}{2}\lambda_{\min}\left(Q\right){\left\|x\right\|}^{2}+\left\|\eta_{0}\right\|\left\|M_{0}^{-1}\left(q\right)\right\|\lambda_{\min}\left(P\right)\left\|x\right\|\\ =-\frac{1}{2}\left\|x\right\|\left[\lambda_{\min}\left(Q\right)\left\|x\right\|-2\left\|\eta_{0}\right\|\left\|M_{0}^{-1}\left(q\right)\right\|\lambda_{\min}\left(P\right)\right] \end{array}$$ (14)

where *λ*_max_(***P***) denotes the maximum eigenvalue of matrix ***P***, *λ*_min_(***Q***) denotes the minimum eigenvalue of matrix ***Q***.

In this case, as long as $\lambda_{\min}\left(Q\right)\geq \frac{2\left\|M_{0}^{-1}\left(q\right)\right\|\lambda_{\max}\left(P\right)}{\left\|x\right\|}\left\|\eta_{0}\right\|$, then: $\dot{V}\leq 0$.

At this time, the system satisfies the stability in the sense of Lyapunov.

According to the stability analysis criteria, if the matrix ***P*** is Symmetric and positive definite, in order to keep the system stable, the Lyapunov equation ***PA*** + ***A*** ^T^ ***P*** = −***Q*** should be satisfied, where ***Q*** ≥ 0.

According to Eq. 2 and Eq. 3 and combining the approximation result $\hat{f}\left(\cdot \right)$ from the neural network, the ultimate controller can be designed as follows:

$$\tau =M_{0}\left(q\right)\left({\ddot{q}}_{d}-k_{v}\dot{e}-k_{p}e\right)+C_{0}\left(q,\dot{q}\right)\dot{q}+G_{0}\left(q\right)-\hat{f}\left(\cdot \right)$$ (15)

**FIGURE 4:**
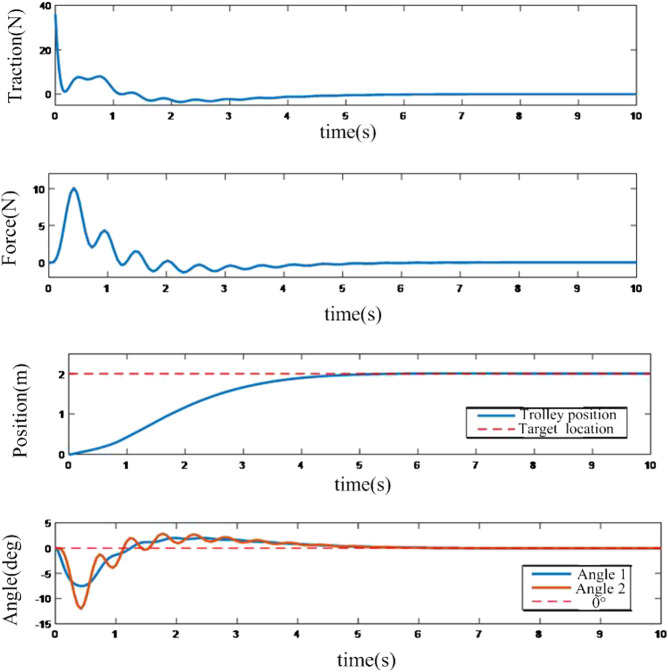
Neural network control response of the control system.

**FIGURE 5:**
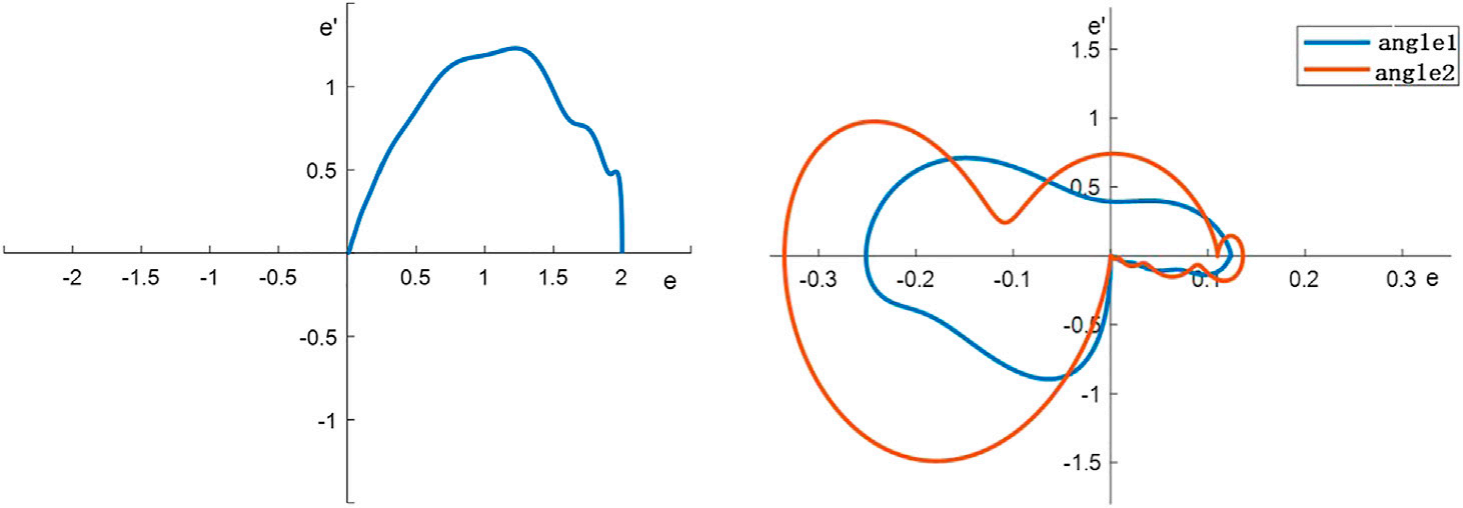
Neural network compensation control system response to phase trajectory.

MATLAB is utilized to establish the crane system and the control system. Suitable control parameters are tuned and the results of the adaptive control system based on neural network compensation are shown in Figure 4 and Figure 5.

It can be depicted from Figure 4 that the neural network compensation can track the position accurately and suppress the swing angle very well. It is also shown in Figure 5 that the final state of the neural network controller is convergent to zero which proved the effectiveness of the controller.

## Experiments

**FIGURE 6:**
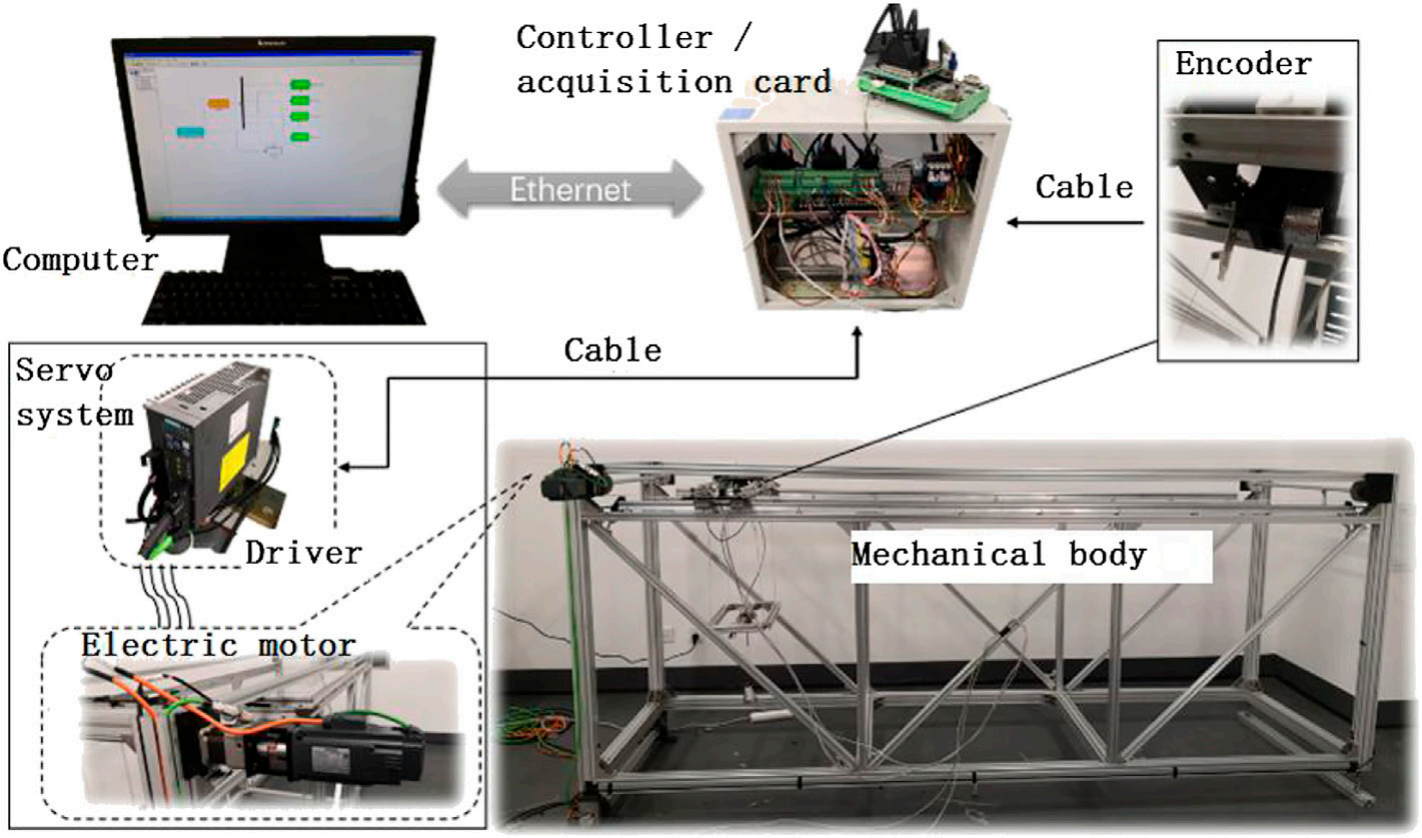
Experimental physical prototype.

The hardware in the loop (HIL) simulation is introduced into the experiment, which is known as an advanced method in scientific research. In this experiment, the HIL is mainly composed of PC, the controller, and the mechanical structure. The PC had already been installed with the MATLAB/Simulink software and was used as a host computer. The motion control card is driven and communicated with the PC. The mechanical structure is driven by the motion card, and the motion parameters of the mechanical structure are collected by the motion control card and passed to the PC. The experimental physical prototype is shown in Figure 6.

In order to testify the performance of the presented neural network-based controller, the well-performed sliding mode controller in (Tuan and Lee, 2013) is also presented as a comparison group. The input of the sliding mode controller is defined as follows:

$$\begin{array}{l} u=-\left(m_{1}+m_{2}\right)l_{1}{\ddot{\theta }}_{1}\cos\theta_{1}-m_{2}l_{2}{\ddot{\theta }}_{2}\cos\theta_{2}+b\dot{x}+\left(m_{1}+m_{2}\right)l_{1}{\dot{\theta }}_{1}^{2}\sin\theta_{1}\\ +m_{2}l_{2}{\dot{\theta }}_{2}^{2}\sin\theta_{2}-\left(M+m_{1}+m_{2}\right)\left(\lambda \dot{x}+\alpha {\dot{\theta }}_{1}+\beta {\dot{\theta }}_{2}\right)-K\mathrm{sgn}\left(s\right) \end{array}$$ (16)

where *K* denotes the amplitude of the control switch gain; *λ*, *α*, *β* represent the designed parameters; sgn(**·**) denotes the sign function.

### Controller Positioning Anti-swing Control Experiments

The sliding mode controller is established in MATLAB/Simulink and the control parameters are tuned to adapt to the experimental facilities. The experimental facility at the original point is static. With the control of the controller, the motion of the trolley follows the target position and the response results are obtained.

**FIGURE 7:**
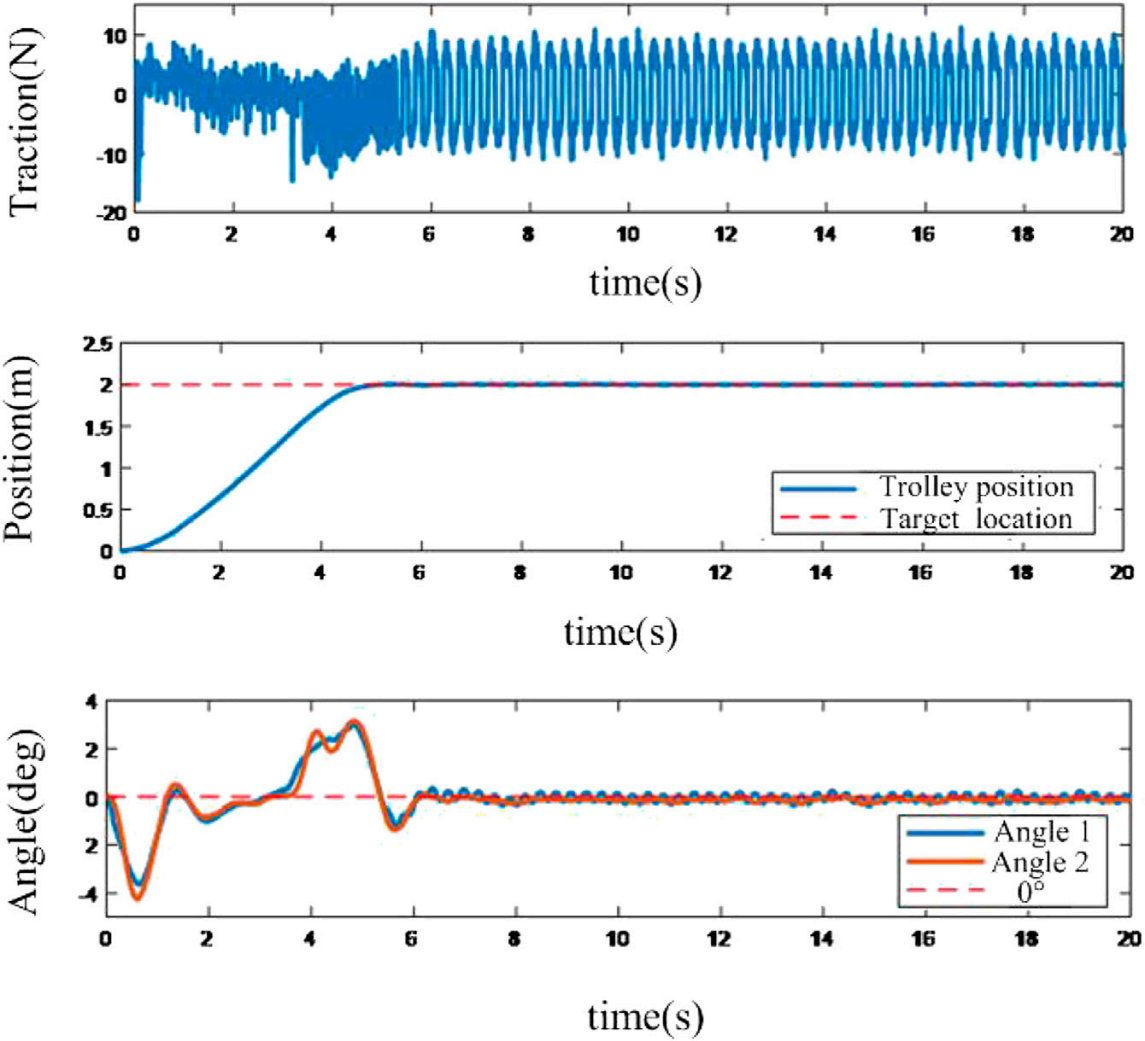
Response of the positioning anti-swing system based on sliding mode method.

It depicts in Figure 7 that the sliding mode control-based method can eliminate the swing angular velocity very quickly in the wide range, and the amplitude of the swing angle is quite small. However, the switch character of the swing angle with high frequency, especially for the swing angles around 0°, will lead to oscillation.

**FIGURE 8:**
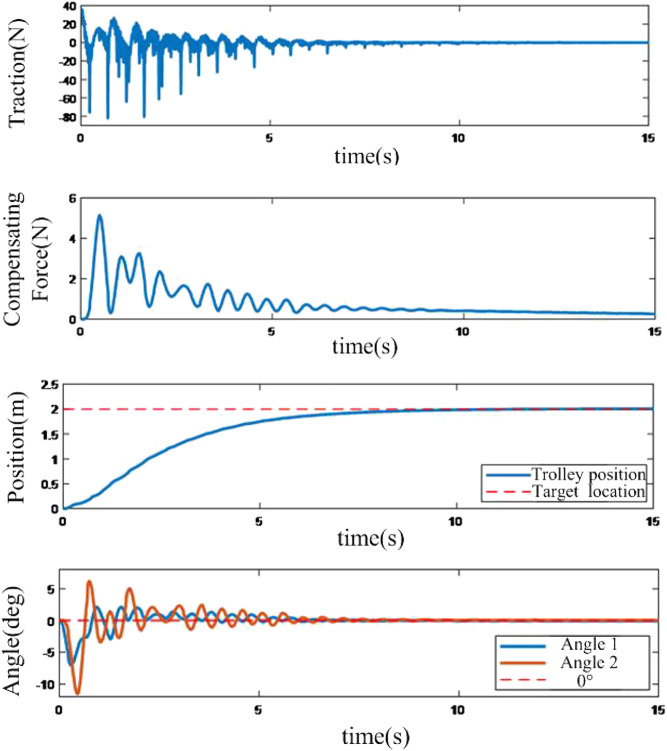
System Response by using neural network compensation adaptive controller.

With the same control object, the results of the RBF neural network compensation controller is shown as follows:

From Figure 8, the adaptive controller based on RBF neural network compensation has no chattering problem of the sliding mode controller, and the process is smoother than the sliding mode controller.

### Anti-disturbance Control Experiment

This experiment is starting with a static trolley and the payload does not have a swing angle. After that, the payload is given a force that makes the payload swing, and the experiment is utilized to evaluate the anti-disturbance performances of the presented controller.

**FIGURE 9:**
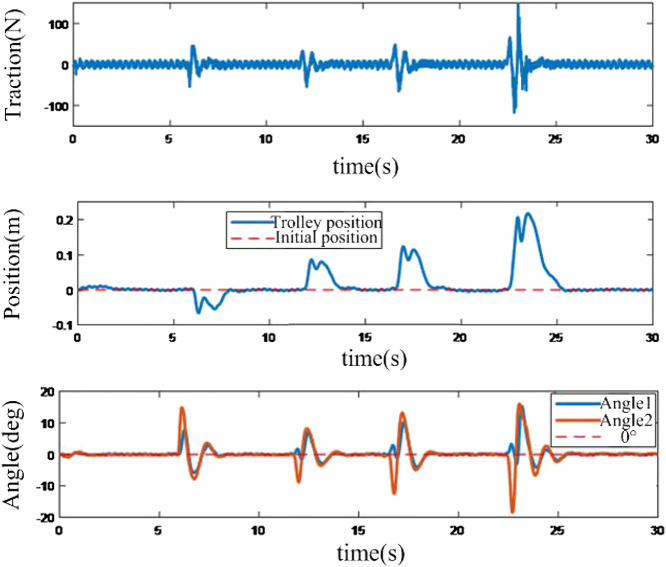
Response of anti-disturbance system based on sliding mode method.

It is shown in Figure 9 that after external excitation the spreader or the payload can go back to the original position very quickly by using a sliding mode controller; the effect of the anti-swing is significant, and the final state is a high-frequency swing state. When the swing angle is big enough, the control effect is better.

**FIGURE 10:**
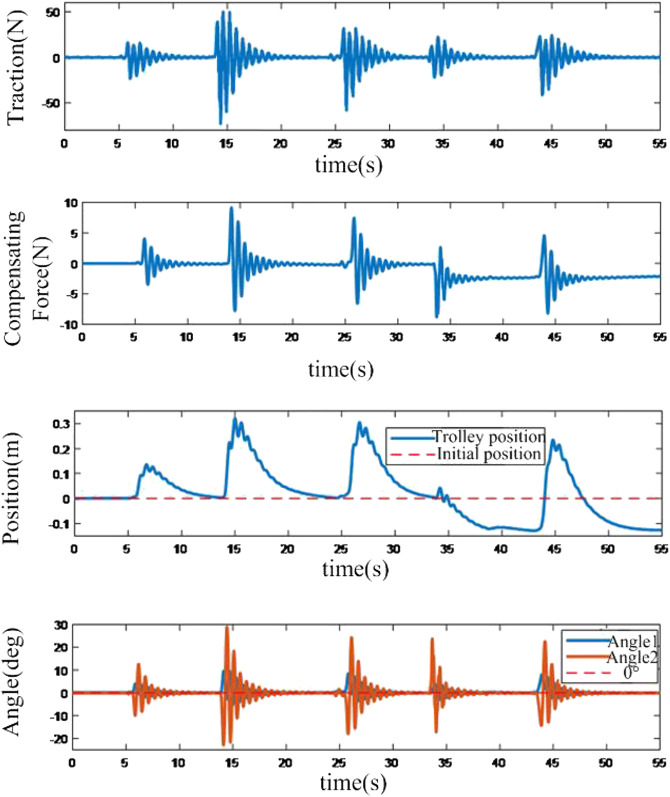
Response of anti-disturbance system based on neural network based compensation controller.

The performance of the neural network-based controller is shown in Figure 10. Compared to the sliding mode-based controller, the neural network-based controller can control a greater swing angle. Additionally, with the bigger excitation, the maximum swing angle can reach 30°. It is proved that the proposed neural network-based controller can control a greater swing angle, which would make a quicker response with the same disturbances, and the working performance is superior in harsh situations.

## Conclusion

The overhead crane with the double-pendulum effect is chosen as the research object in this paper. A dynamic model of the crane has been established first. Then, an RBF-based neural network compensation adaptive controller is proposed, and simulations with MATLAB are also performed to verify the stability of the system. Finally, experiments with HIL are presented to test the presented controller where a comparison with a sliding mode based controller is also presented. The experimental results show that compares with the traditional sliding mode based controller, the neural network-based controller has a superior performance and better ability to anti-disturbances and without oscillation. Moreover, the proposed controller is also proved to be adequate for real-time control by experimental results.

## Data Availability

The original contributions presented in the study are included in the article/Supplementary Material, further inquiries can be directed to the corresponding author.
